# Thirty synchronous medullary and papillary thyroid carcinomas

**DOI:** 10.3389/fendo.2023.1153248

**Published:** 2023-03-31

**Authors:** Daqi Zhang, Mingyu Yang, Xin Zhang, Cheng Wang, Kunlin Li, Hongbo Wang, Hao Chi, Chengqiu Sui, Gianlorenzo Dionigi, Hui Sun

**Affiliations:** ^1^Division of Thyroid Surgery, Jilin Provincial Key Laboratory of Surgical Translational Medicine, Jilin Provincial Precision Medicine Laboratory of Molecular Biology and Translational Medicine on Differentiated Thyroid Carcinoma, China-Japan Union Hospital of Jilin University, Changchun, Jilin, China; ^2^Division of Pathology, China-Japan Union Hospital of Jilin University, Changchun, Jilin, China; ^3^Department of Pathophysiology and Transplantation, University of Milan, Milan, Italy; ^4^Division of Surgery, Istituto Auxologico Italiano Instituto di Ricovero e Cura a Carattere Scientifico (IRCCS), Milan, Italy

**Keywords:** thyroid, mixed tumor, medullary thyroid carcinoma, papillary thyroid carcinoma, clinical features, surgery

## Abstract

**Synopsis for table of contents:**

An exceptional number of synchronous MTC/PTC in the same thyroid gland is presented. This may be the most numerous case series reported in the literature. Synchronous PTC/MTC in the same thyroid gland were classified into 4 subtypes and the clinical and pathological aspects as well as the results are presented.

**Background and objectives:**

The synchronous occurrence of multiple neoplastic processes in the thyroid gland is unusual. We investigated the clinicopathological features of 30 medullary thyroid carcinomas (MTC) in association with papillary (PTC).

**Method:**

Retrospective analysis of operated patients for thyroid tumors. Synchronous PTC/MTC in the same thyroid gland were classified into 4 subtypes: (type I) True mixed MTC/PTC, MTC and PTC closely intermingled. (Type II) Collision MTC/PTC, i.e. tumors that meet at the same site, invade each other and appear as a single mass in the thyroid gland, i.e. MTC and PTC merge. (Type III) Synchronous anatomically separate tumors in the same thyroid lobe, i.e. separated from each other by non-tumorous thyroid parenchyma. (Type IV) Synchronous tumors occurring in separate anatomical lobes or in the isthmus. Clinical and pathological data were reviewed. Location: Department of thyroid surgery, China-Japan Union Hospital, Jilin University. Time frame: 14 years (June 2008-November 2022).

**Results:**

Thirty patients were identified with an overall prevalence of 28621 (0.1%). 17 (56.7%) were male, 13 (43.3%) female, mean age 51.3 ± 11.0 years, mean BMI 23.6 ± 3.6kg/m^2^. Mean duration of symptoms was 11.2 ± 18.4 months. Mean calcitonin level was 133.7 ± 196.4 pg/ml. Fine needle aspiration (FNA) was offered in 21 cases: 9 (42.9%) were suspected carcinoma, 9 (42.9%) PTC, 1 (4.8%) MTC, 2 (9.4%) MTC/PTC. Pathology revealed type I 4 (13.3%), type II 2 (6.7%), type III 14 (46.7%), type IV 10 (33.3%). The mean diameter of MTC was 1.6 ± 2.0cm, 18 (60%) were micro-MTC. The mean diameter of PTC was 0.9 ± 1.9 cm, 26 (86.7%) were micro-PTC. In 16 (53.3%) micro-PTC/-MTC occurred in synchronous sequence. Four patients had a recurrence: 2 had to be re-operated due to MTC recurrence, 2 died due to distant metastases (bone, liver).

**Conclusion:**

We report an exceptional number of MTC/PTC in the same thyroid gland. This may be the most numerous case series reported in the literature. The clinical and pathological aspects as well as the results are presented.

## Introduction

Papillary thyroid carcinoma (PTC) accounts for more than 90% of all thyroid carcinomas and originates from the follicular epithelial cells (1). Medullary thyroid carcinoma (MTC) is a tumor that originates from the parafollicular cells, the C cells. MTC produces calcitonin, and an elevated calcitonin level is a major feature of this tumor. PTC and MTC have a different prognosis (1).

The simultaneous occurrence of PTC and MTC is rare and accounts for less than 1% of tumors (2–4). The synchronous occurrence of these two carcinomas occurs either as discrete lesions or as a mixed lesion (2–4).

We retrospectively studied the clinical profiles and pathological features of 30 combined PTC and MTC diagnosed over a 14-year period.

## Patients and methods

### Study design

Retrospective study.

### Setting

Thyroid surgery department, China-Japan Union Hospital, Jilin University. Hospital volume is categorized based on > 4,000 thyroid surgeries performed annually.

### Ethics

No ethics committee review is foreseen for retrospective studies.

### Time frame

June 2008 to November 2022 (period of 14 years).

### Patients eligibility

Inclusion criteria (1) Proven postoperative paraffin pathology of MTC combined with PTC; (2) patients who underwent thyroid surgery for the first time; (3) surgery offered in our department; (4) complete follow-up; (5) complete clinical data; (6) pathological examination offered in our hospital.

Exclusion criteria (1) did not meet the criteria of synchronous thyroid tumor; (2) other pathological types of thyroid cancer; (3) combination with other malignant tumors of the head and neck; (4) reoperation; (5) palliative surgery; (6) cases with insufficient histological material; (7) follow-up < 3 months; (8) unknown survival status.

### Registry and database

The Department of Thyroid Surgery database is a registry required by law to record all newly diagnosed thyroid cancer cases. It is under the auspices of the China-Japan Union Hospital of Jilin University.

### Definitions

*FNA.* According to the Bethesda classification for fine needle aspirations of thyroid nodules (5).

*PTC and MTC.* Defined by ATA guidelines and the Joint Committee of Cancer (AJCC 8th edition) (1, 6).

*Micro-PTC and micro-MTC.* Defined by the largest extension of 1.0 cm or less (1, 6).

Multifocal tumor. Multifocal carcinomas may occur as microcarcinomas or larger tumors at two or more sites in the gland (1, 6).

**Figure 1:**
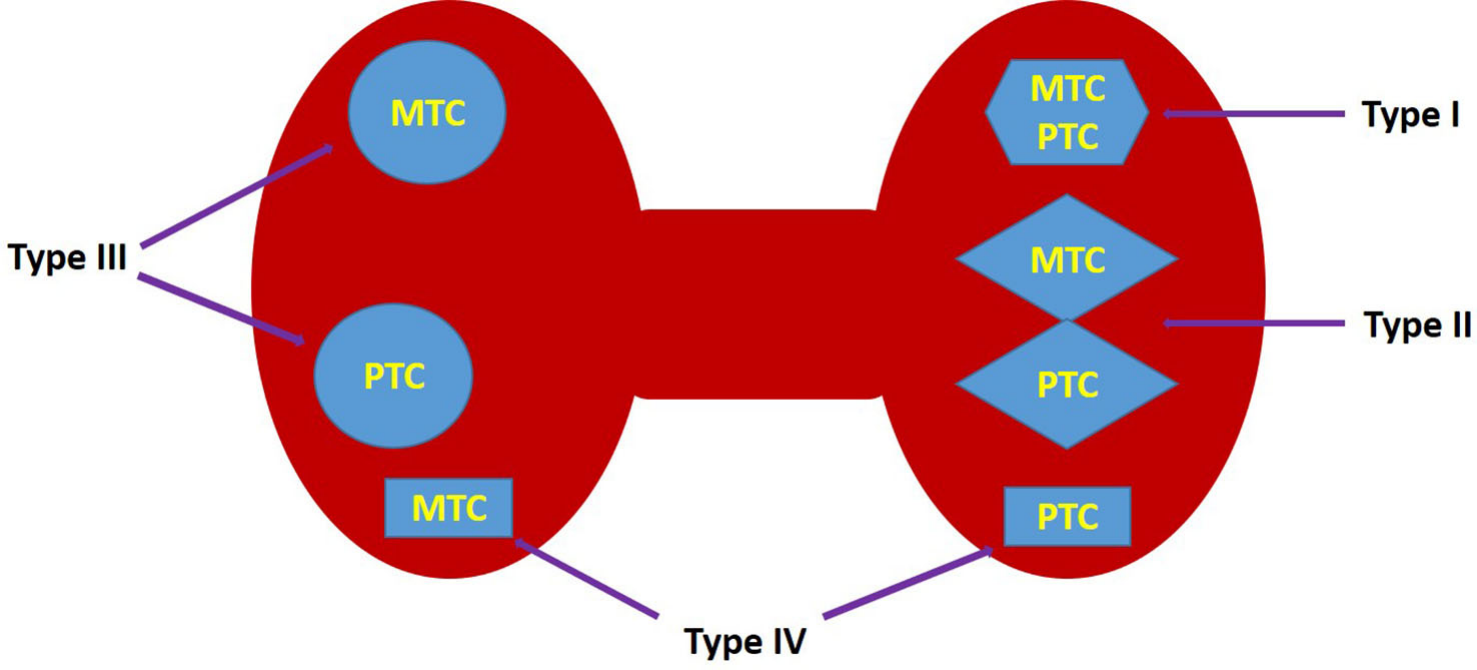
Synchronous tumors were divided into 4 subtypes.

**Figure 2:**
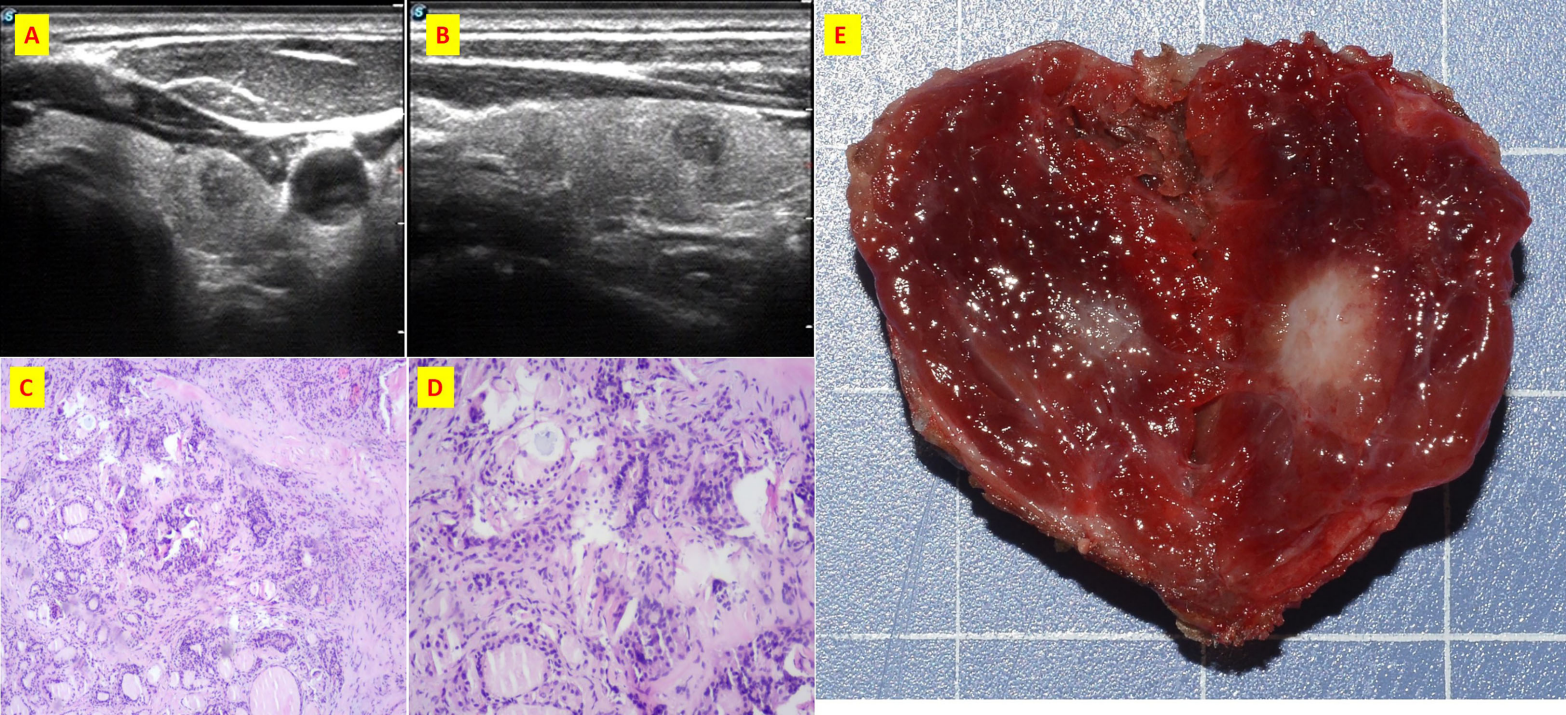
The patient n.2 that MTC and PTC was located in the same tumor. **(A)** ultrasound of transverse; **(B)** ultrasound of longitudinal; **(C)** Histological sections of HE x40; **(D)** Histological sections of HE x100; **(E)** Intraoperative resection of tumor profile.

**Figure 3:**
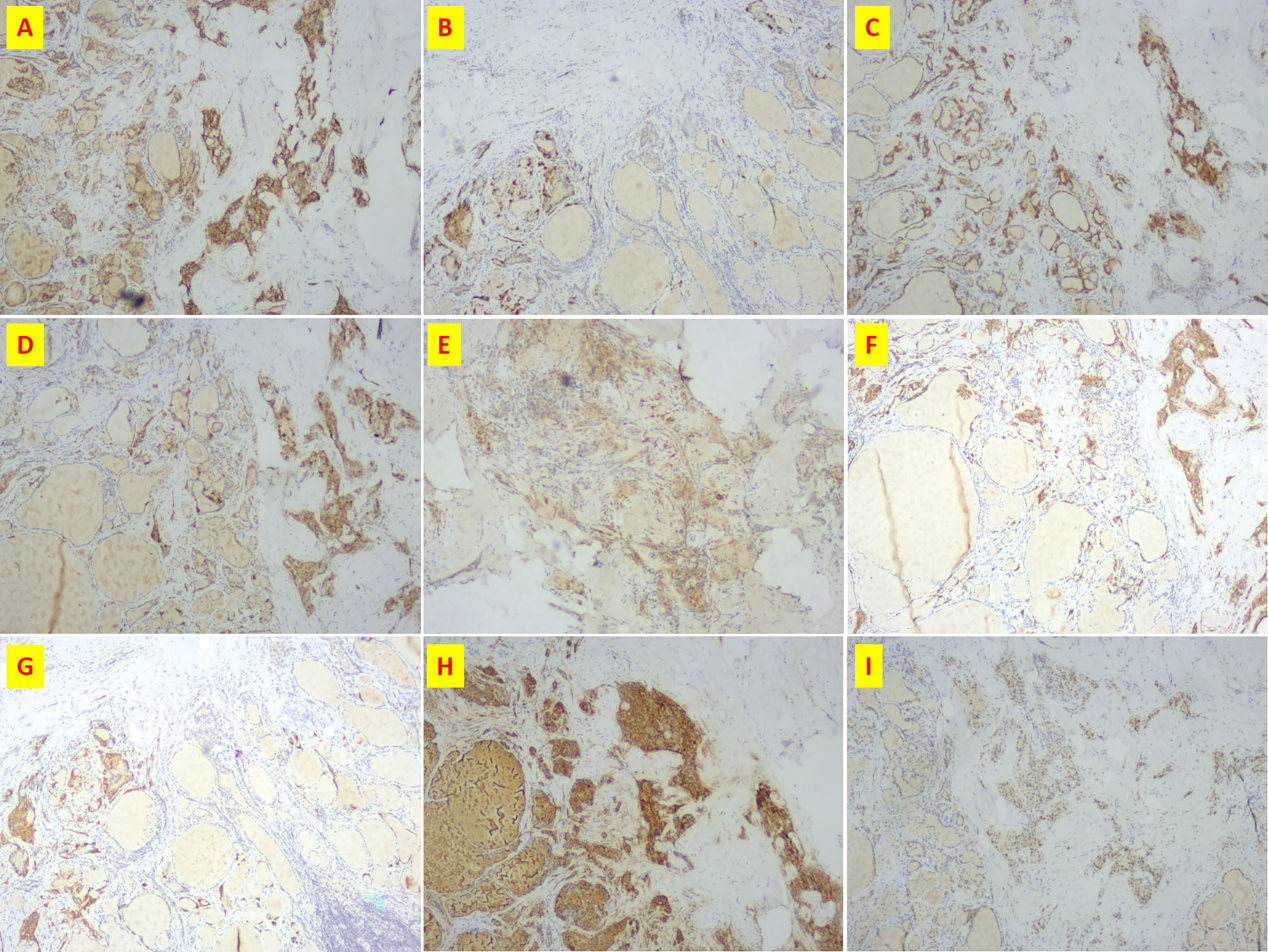
Immunohistochemistry of patient n.2 that MTC and PTC was in the same lesion. **(A)** CD56(+); **(B)** CgA(+); **(C)** CK19(+); **(D)** CT(+); **(E)** Galectin3(+); **(F)** Syn(+); **(G)**Syn(+); **(H)** Tg(+); **(I)**TTF-1(+).

**Figure 4:**
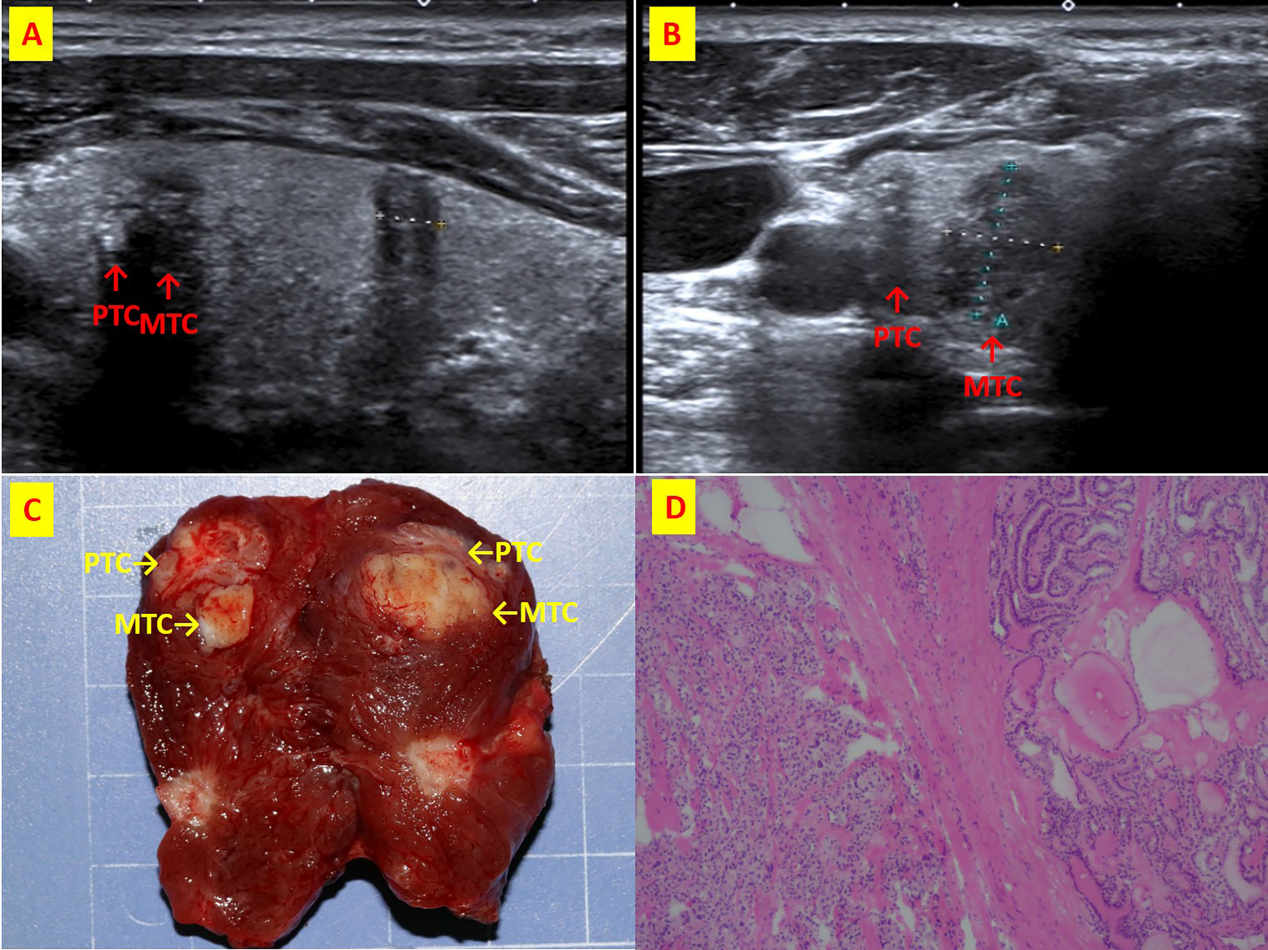
The patient n.6 that MTC and PTC are located in the right thyroid lobe closely. **(A)** ultrasound of longitudinal; **(B)** Ultrasound of transverse; **(C)** Intraoperative resection of tumor profile; **(D)**Histological sections of HE x40.

**Figure 5:**
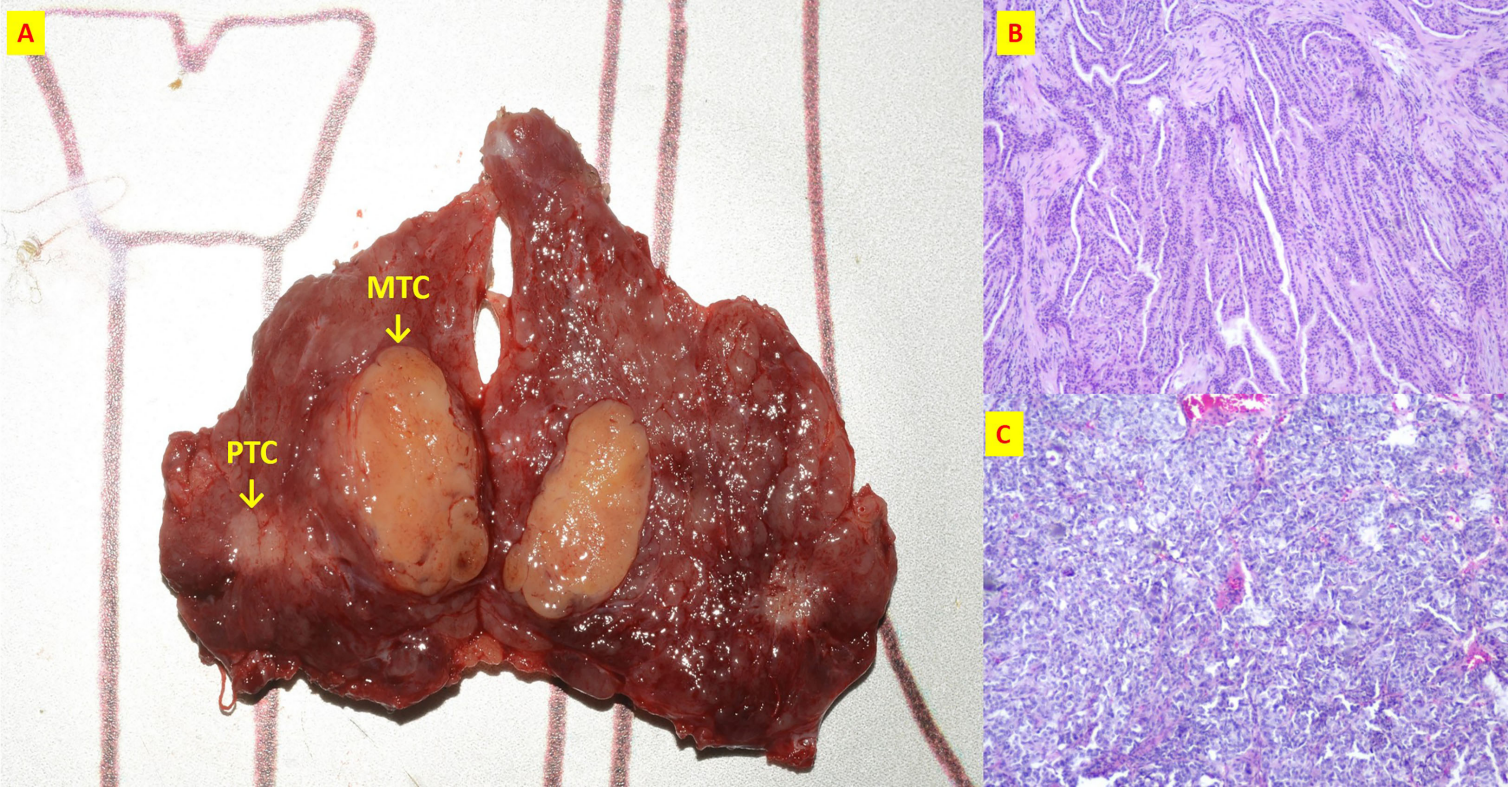
The patient n.17 that MTC and PTC were located in the different tumor of ipsilateral lobe. **(A)** Intraoperative resection of tumor profile; **(B)** Histological sections of HE x40 of PTC; **(C)** Histological sections of HE x40 of MTC;.

**Figure 6:**
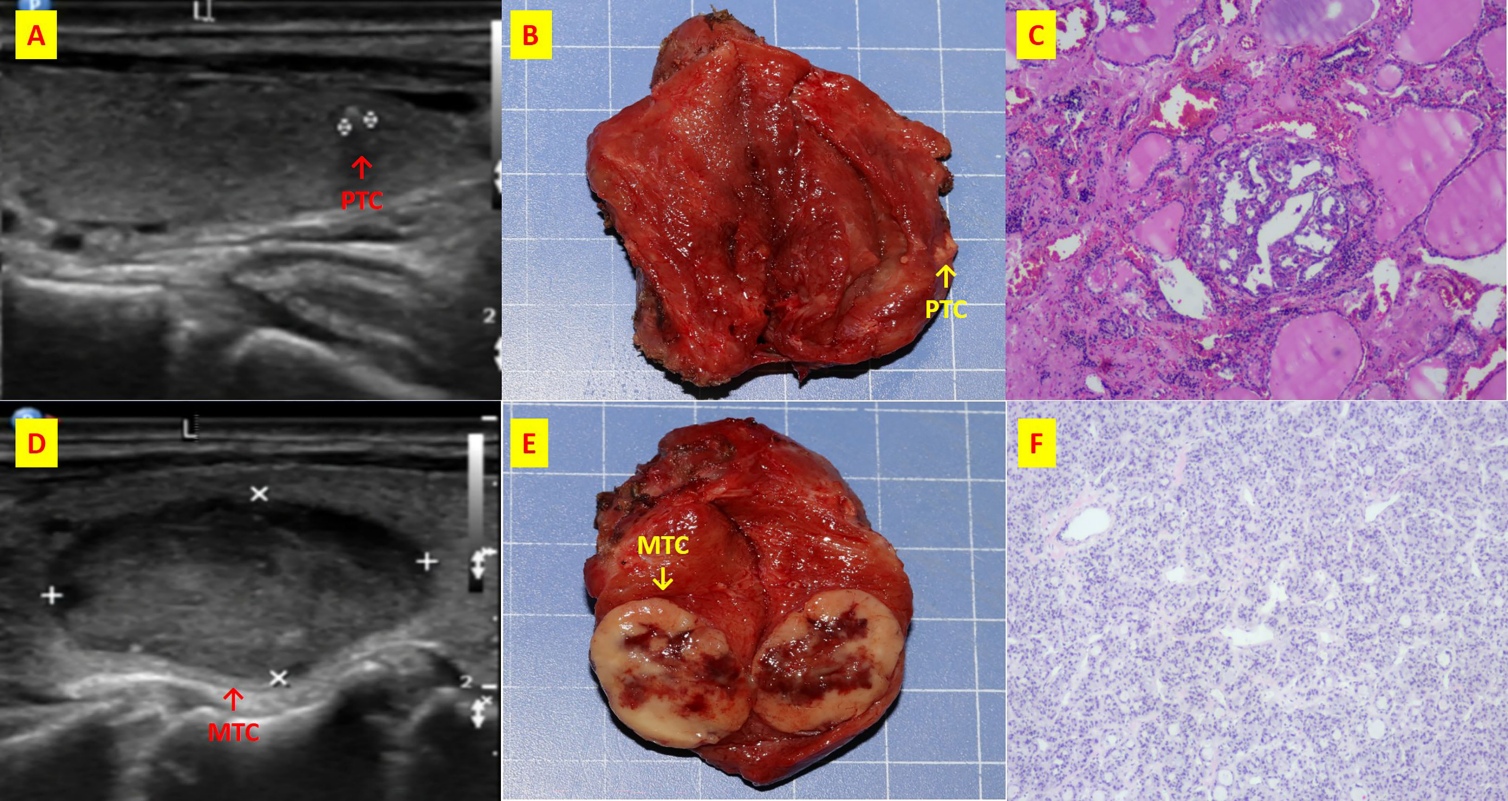
The patient n.26 that MTC and PTC are located in the different thyroid lobe respectively. **(A)** ultrasound of longitudinal of right PTC; **(B)** Intraoperative resection of PTC tumor profile; **(C)** Histological sections of HE x40 of PTC; **(D)** ultrasound of longitudinal of left MTC; **(E)** Intraoperative resection of MTC tumor profile; **(F)** Histological sections of HE x40 of MTC;.

*Sinchronic thyroid tumors.* Defined by Sung CT and Sadow P as tumors consisting of two histologically distinct neoplasms that meet in the same thyroid gland, i.e. one MTC is accompanied by a second tumor component derived from the follicular epithelial cells (7, 8).. Synchronous tumors have been divided into 4 subtypes (**Figure 1**):

1. Type I: True mixed MTC/PTC, i.e. MTC and PTC that are closely intertwined; this is also referred to as true mixed medullary-follicular carcinoma (**Figures 2**, **3**)
2. Type II. Collision MTC/PTC, i.e. tumors consisting of two histologically distinct neoplasms that meet at the same site, invade each other and appear as a single mass in the thyroid gland, i.e. MTC and PTC merge. (**Figure 4**, **Supplementary Figure 1**)
3. Type III. Synchronous, anatomically separate tumors in the same thyroid lobe separated by non-tumor thyroid parenchyma. (**Figure 5**, **Supplementary Figure 2**) (Video 1)
4. Type IV. Synchronous tumors occurring in separate anatomical lobes or in the isthmus. (**Figure 6**, **Supplementary Figure 3**)

*Calcitonin (Ctn).* Normal Ctn levels 0.15~6.00 ng/L in females and 0.15~9.20 ng/L in males.

*Carcinoembryonic antigen (CEA).* The reference range is 0.5-5ng/ml blood.

*Surgical procedures.* Consensus statement on terminology and classification of central neck dissection in thyroid cancer (9).

*Ultrasound (US).* Ultrasound examination was performed with high-frequency probes (at least 13 MHz). All thyroid nodules were assessed by a physician with approximately 10 years of experience in thyroid medicine. The optimal settings were always made in the same order (gain, field of view, magnification) to obtain ideal measurements of all thyroid nodules examined. The nodule was assessed by measuring the long axis on US scan on greyscale images (1, 6).

*TNM staging.* Staging was performed in all cases by US examination and in histopathological cases where cervical lymph node dissection had been performed (1, 6). Thyroid cancer is staged according to the most advanced PTC or MTC (1, 6).

The lymph node ratio is defined as the number of positive lymph nodes divided by the total number of excised lymph nodes (9, 10).

### Identification of synchronous MTC/PTC and pathology

Surgically resected tissues were fixed in 10% neutral buffered formalin and embedded in paraffin (1, 6). 5 μm thick sections were cut and stained with haematoxylineosin, Congo red and periodic acid-Schiff (1, 6). Immunohistochemical studies were performed on 5 μm thick paraffin sections using a strepavidinbiotin-peroxidase-conjugated detection system. The specificity of immunostaining was checked by replacing the primary antisera with normal rabbit serum or with non-immune mouse ascites in the case of monoclonal antibodies (1, 6).

### Follow-up

All patients were followed up at the Department of Thyroid Surgery, China-Japan Union Hospital, Jilin University. Follow-up for all patients included (1) measurements of Ctn, thyroglobulin, TSH and free thyroxine under suppressive therapy and (2) annual ultrasound examination of the neck (1, 6). Diagnostic whole-body scans and determination of thyroglobulin after discontinuation of thyroxine were performed 9-12 months after treatment with 131I and as needed thereafter (those treated with I131) (1, 6).

### Endpoints of the study

The database was searched for synchronous MTC and PTC using keywords such as “medullary AND papillary AND thyroid’, ‘mixed thyroid’, ‘mixed medullary AND papillary’, ‘collision tumor”, ‘synchronous’. Clinical data were taken from the electronic medical record. Imaging data were taken from the image archive. We analyzed clinical manifestations, laboratory tests, imaging, preoperative diagnosis, surgical procedures and pathological findings.

### Statistical analysis

P < 0.05 was considered statistically significant, and all P values were two-sided. Kaplan-Meier survival analysis followed by log-rank test was performed. Other statistical analyses were performed using SPSS software version 23.0 (IBM Corp. Released 2015. IBM SPSS Statistics for Windows, version 23.0. Armonk, NY: IBM Corp).

## Results

### Demographic data of the patients

During the 14-year analysis of this study, we operated on 28621 thyroid tumors. Among these 28621 thyroid tumors, 30 synchronous MTC/PTC tumors were identified in our database (**Table 1**). This corresponds to an incidence of 30/28621 (0.1%).

**Table 1:** Preoperative basic information of 30 patients.

Case	Age (years)	Gender	BMI	FNA	Tumor diameter (cm)			Ctn	CEA	Operation
					PTC	MTC		0.5-6pg/ml	0-5ng/ml	
Type I										
1	51	M	27.7	MTC	2.0			14.3	18.8	TT+CLND+BLND
2	30	M	22.5	SC	0.4			2.56	–	TT+CLND
3	52	F	18.8	PTC	0.7			7.19	4.6	TT+CLND
4	76	F	24.2		11			0.15	–	TT+CLND
Type II										
5	43	F	27.3	PTC	0.4	0.1		44.06	–	L+CLND
6	58	M	33.8	–	0.7	1.6		92.2	15.3	TT+CLND
Type III										
7	58	F	25.7	SC	0.2	1.3		81.1	–	TT+CLND+LLND
8	44	M	23.3	–	0.6	2.5		214.63	–	TT+CLND+LLND
9	58	M	21.6	PTC	0.3	3		0.59	–	TT+CLND+LLND
10	53	M	20.7	SC	0.1	1		26.3	5.5	TT+CLND+LLND
11	49	F	22.5	PTC	0.7	0.4		1.08	–	L+CLND
12	59	M	33.4	–	1	0.8		20	–	TT+CLND
13	55	F	23.3	PTC+MTC	1	0.7		120.84	5	TT+CLND
14	46	F	22.8	–	0.2	0.2		2.33	0.5	TT+CLND
15	41	F	20	SC	0.6	0.6		33.57	4.8	TT+CLND
16	48	M	22.8	PTC	0.9	0.5		2.56	–	TT+CLND
17	58	F	23.4	PTC	0.8	1.5		219	33.8	TT+CLND+LLND
18	49	M	23.9	SC	0.2	0.8		11.41	–	TT+CLND
19	49	M	23.7	SC	0.5	3.5		585	2.7	TT+CLND+BLND/CLND
20	62	F	21.5	PTC	0.5	0.3		14.8	2.5	TT+CLND
Type IV										
21	53	F	23.8	–	0.4	0.9		202.84	–	TT+CLND
22	57	M	20.7	–	0.15	2		585	–	TT+CLND+LLND
23	43	M	21.2	SC	1.8	0.6		23.6	–	TT+CLND+BLND
24	31	F	17.2	PTC	1.1	0.4		0.59		TT+CLND+LLND
25	68	M	28.1	–	0.2	4.5		585	356.5	TT+CLND+LLND
26	67	M	18.8	PTC+MTC	0.6	2.5		585	145.5	TT+CLND+LLND
27	52	M	22.3	SC	0.05	0.74		3.64	–	TT+CLND
28	50	F	24.9	PTC	0.4	0.3		1.43	1.96	TT+CLND
29	56	M	24.2	SC	0.04	0.6		184.43	5.9	TT+CLND+LLND
30	22	M	23.2	–	0.1	2.5		347	6.5	L+CLND/L+CLND+LLND

M, Male; F, Female; PTC, Papillary thyroid carcinoma; MTC, Medullary thyroid carcinoma; SC, Suspicious cancer; TT, Total Thyroidectomy; L, lobectomy; CLND, Central lymph node dissection; LLND, Lateral lymph node dissection; BLND, Bilateral lymph node dissection. –,none

### Report of clinical characteristics

The patients were all Chinese. The mean age was 51 ± 11 years. There were no pediatric patients in this series. 17 cases (56.7%) were male, 13 (43.3%) were female, the male to female ratio was 1.3:1. The mean BMI was 23.6 ± 3.6 kg/m2 (3 patients had a BMI > 28 kg/m2). The mean duration of symptoms was 11.2 ± 18.4 months. 27 of the 30 cases (90%) had thyroid swelling, 3 (10%) suffered from dysphagia and dyspnea.

### Thyroid function test

All patients were euthyroid at the time of surgery. Mean values of TSH and Tg were 2.69 ± 1.73mIU/L and 13.86 ± 21.16ng/ml, respectively.

### US examinations

All patients underwent US examination before surgery. Four patients (patient no.1, 2, 3, 4) were type I. All of them had hypoechoic nodules. Irregular shaped thyroid nodules were found in 3 cases (75%). Colour Doppler ultrasonography showed increased blood flow within the nodule in one patient (25%), patchy vascularity in one (25%) and any pattern in two (50%). 2 (50%) had microcalcifications.

Of the remaining 26 patients, 23 had PTC lesions found on ultrasound. There were 18 hypoechoic nodules (60%), 4 (13.3%) with mixed echo and 1 (3.3%) with hyperechoic. Irregular shaped thyroid nodules were found in 21 cases (70%). 5 patients (16.7%) had increased intranodular blood flow, 2 (6.7%) had patchy vascularity, 16 (53.3%) had undetectable vascularity. 8 (26.7%) had microcalcifications.

Of the 26 patients, all had MTC lesions found under US. 18 cases (59.3%) were echo poor, 8 (26.7%) had mixed features. An irregular shaped thyroid nodule was found in 1 case (3.3%). 11 (36.7%) had increased intranodular blood flow, 2 (6.7%) were patchy, 13 (43.3%) had no vascularity. 9 (30%) had microcalcifications.

### Cytological findings

**Figure 7:**
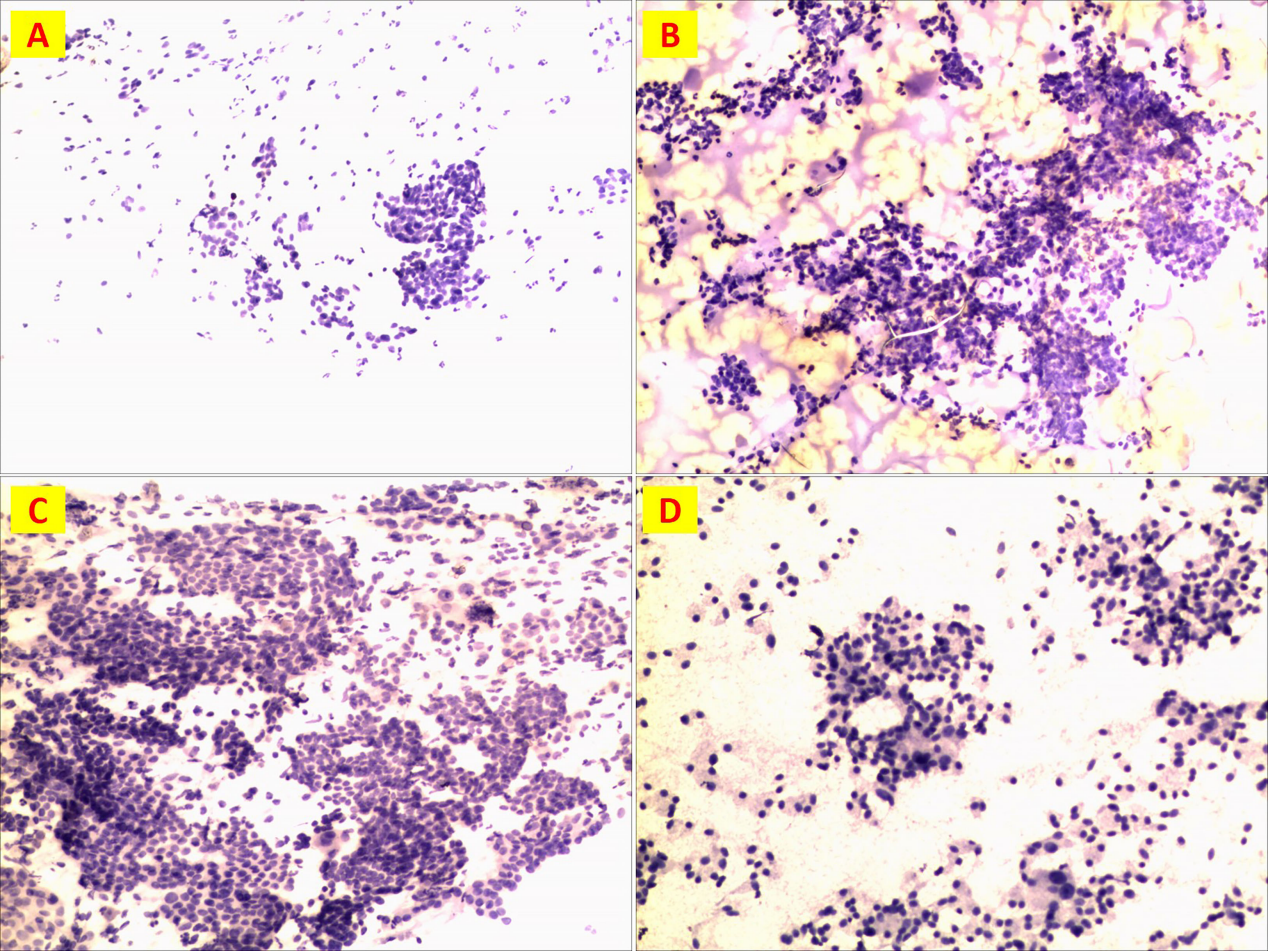
Preoperative FNA detection. **(A)** Patient n.18 suspicious cancer; **(B)**Patient n. 5 PTC; **(C)**Patient n. 26 PTC; **(D)**Patient n. 26 MTC.

A total of 21 cases were examined by FNA before surgery, including 9 (42.9%) with suspected carcinoma, 9 (42.9%) with suspected PTC, 1 (4.8%) MTC and 2 (9.4%) MTC in combination with PTC. (**Figure 7**) In detail, FNA was performed in 21 patients out of 30. 8 patients were selected based on pre-operative high Ct levels. While Patient 14 (normal pre-operative Ct) rejected preoperative FNA on a suspicious nodule on ultrasound

### Characteristics of the treatments

Unilateral lobectomy + N-dissection of the central compartment was offered in 3 (10%). 14 (46.7%) total thyroidectomy + central N dissection. Total 10 (33.3%) thyroidectomy + central and unilateral N dissection. 3 (10%) total thyroidectomy + central & bilateral N dissection (**Table 1**). 2 (6.7%) cases (patient no.1, 25) received postoperative RAI treatments.

### Classification and gross pathological findings

Stratification of synchronous tumors was: type I 4 (13.3%), type II 2 (6.7%), type III 14 (46.7%), type IV 10 (33.3%).

MTC were located in the left lobe in 14 cases (46.7%), in the right lobe in 16 cases (53.3%). PTC lesions were located in the left lobe in 11 cases (36.7%), in the right lobe in 13 cases (43.3%) and in both lobes in 6 cases (20%). Overall, 16 (53.3%) tumors were ≤ 1 cm in diameter, 7 (23.3%) were 1-2 cm in diameter, 5 (16.7%) were 2-4 cm in diameter and 2 (6.7%) were > 4 cm in diameter.

MTC diameter was 0.1~11.0cm, mean 1.6 ± 2.0 cm, 18 cases (60%) were micro-MTC. PTC diameter was 0.04-11.0 cm, average 0.9 ± 1.9 cm, 26 (86.7%) were micro-PTC. 16 (53.3%) were both micro-MTC and PTC.

**Figure 8:**
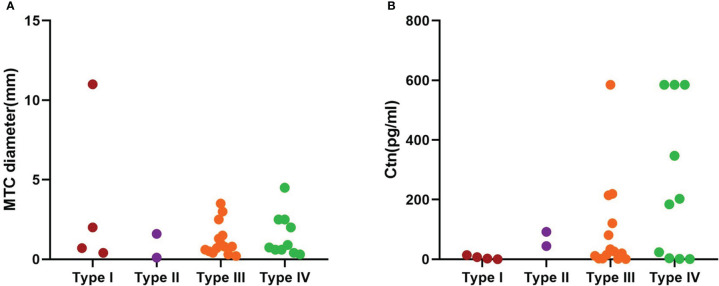
MTC tumor diameter and Ctn in each subtype. **(A)** MTC tumor diameter; **(B)**Ctn.

The average tumor size was graded as follows: Type I 3.5 ± 5.0 cm, Type II: MTC 0.9 ± 1.1 cm and PTC 0.6 ± 0.2 cm, Type III: MTC 1.2 ± 1.0cm and PTC 0.5 ± 0.3cm, Type IV MTC 1.5 ± 1.4cm and PTC 0.5 ± 0.6cm. (**Figure 8A**)

Gross T infiltration of extrathyroidal tissue occurred in 10 (33.3%) cases. Gross N-metastases occurred in 1 case (3%): The diameter of the N metastases was larger than that of the T lesions (the diameter of the MTC lesions was 2 cm and the maximum diameter of the N metastases was 3.5 cm (patient no. 22).

### Micro-pathological T findings

Immunohistochemical examination of the MTC in 24 patients revealed 66.7% CT, 80% synaptophysin (Syn) in 25 patients, 87.5% chromogranin A (CgA) in 24 patients, 100% Thyroid transcription factor 1 (TTF-1) in 25 patients, 81.8% Congo red staining in 21 patients and 44% thyroglobulin (Tgin) 25 patients (**Table 2**)

**Table 2:** Information on immunohistochemical testing of the patient’s MTC lesions.

Immunohistochemistry Testing Program	Test cases	Number of positive cases	Positive percentage (%)
CT Syn CgA TTF-1 Congo red Tg	24 25 24 25 11 25	16 20 21 25 9 11	66.7 80 87.5 100 81.8 44

There were 0 (0%) multifocal MTC. 9 patients (30.0%) had multicentric PTC (patient nos. 3, 6, 7, 16, 17, 18, 20, 24, 30).

Micro-T infiltration of the thyroid capsule, extrathyroidal tissue and vessels occurred in 0 (0%) vs. 3 (10%), 1 (3.3%) vs. 4 (13.3%), 1 (3.3%) vs. 1 (3.3%) MTC and PTC, respectively.

### N status

The mean number of resected N was 14.7 ± 15.3. The mean number of metastatic N was 3.1 ± 8.5. 15 (50%) patients had N metastases, specifically: 9 patients (60%) had N metastatic PTC, 4 patients (26.7%) had MTC N, 2 patients (13.3%) had both MTC and PTC N metastatic. Further information can be found in **Supplementary Table 1**.

### M status

No patient had distant metastases at the time of surgery.

### Dominant tumor

The tumor with the highest TNM status was MTC in 15 (50%) cases and PTC in 14 (46.7%) cases. In 1 (3.3%) case (patient no. 8) the tumor was dominated by MTC and N was PTC.

Stratified by MTC risk, type I was mainly stage III, which accounted for 75%, all types II were stage II, type III was mainly stage I, which accounted for 64.3%,

Type IV was mainly stage IVA, which accounted for 40%. (**Table 3**).

**Table 3:** The MTC risk stratification in synchronous tumors of 4 subtypes.

	I	II	III	IVA	IAC
**Type I**	**0**	**0**	**3** (Patient n.2, 3, 4)	**0**	**1** (Patient n.1)
**Type II**	**2** (Patient n.5, 6)	**0**	**0**	**0**	**0**
**Type III**	**9** (Patient n.8, 10, 12, 13, 14, 16, 17, 18, 20)	**1** (Patient n.9)	**3** (Patient n.11, 15, 19)	**1** (Patient n.7)	**0**
**Type IV**	**2** (Patient n.21, 27)	**1** (Patient n.26)	**2** (Patient n.28, 30)	**4** (Patient n.22, 23, 24, 29)	**1** (Patient n.25)

### Dynamic Ctn

All patients underwent a preoperative Ctn test. 21 patients had elevated calcitonin levels (70%), averaging 133.7 ± 196.4pg/ml (**Table 4**). 9 (30%) had normal Ctn levels before and after surgery. Stratification of Ctn levels (mean) in synchronous tumors was type I 6.0 ± 6.2 pg/ml, type II 68.1 ± 34.0 pg/ml, type III 95.2 ± 160.0 pg/ml, type IV 251.9 ± 255.7 pg/ml (**Figure 8B**).

**Table 4:** Relationship between calcitonin changes and clinicopathological features.

	Preoperative calcitonin elevated			Preoperative calcitonin normal		
	Postoperative cure	Postoperative stability	Postoperative recurrence	Postoperative cure	Postoperative stability	Postoperative recurrence
**Tumor diameter of MTC**	**16** (Patient n.3, 5, 6, 7, 8, 10, 12, 13, 15, 17, 18, 20, 21, 22, 23, 26)	**1** (Patient n.29)	**4** (Patient n.1, 19, 25, 30)	**9** (Patient n.2, 4, 9, 11, 14, 16, 24, 27, 28)	**0**	**0**
≤1cm	**10** (Patient n.3, 5, 10, 12, 13, 15, 18, 20, 21, 23)	**1** (Patient n.29)	**0**	**7** (Patient n.2, 11, 14, 16, 24, 27, 28),	**0**	**0**
1-2cm	**4** (Patient n.6, 7, 17, 22)	**0**	**1** (Patient n.1)	**0**	**0**	**0**
2-4cm	**2** (Patient n.8, 26)	**0**	**2** (Patient n.19, 30)	**1** (Patient n.9)	**0**	**0**
≥4cm	**0**	**0**	**1** (Patient n.25)	**1** (Patient n.4)	**0**	**0**
**Tumor invasion of MTC**	**0**	**0**	**2** (Patient n.1, 25)	**0**	**0**	**0**
**Lymph node metastasis of MTC**	**1** (Patient n.22)	**1** (Patient n.29)	**4** (Patient n.1, 19, 25, 30)	**0**	**0**	**0**

Postoperative cure: Postoperative calcitonin was within the normal range, and there were no recurrent lesions;

Postoperative stability: Postoperative calcitonin was exceeded the normal range and remained at a high level, and there were no recurrent lesions;

Postoperative recurrence: Postoperative calcitonin was exceeded the normal range, and there were recurrent lesions.

### Dynamic CEA

CEA was tested before surgery in 15 patients with a mean value of 40.6 ± 94.6 ng/ml (**Table 5**). In 9 cases (60%) the CEA level was elevated. In these 9 cases, the synchronous tumors were stratified as follows: Type I 1 (11.1%), Type II 1 (11.1%), Type III 3 (33.3%), Type IV 4 (44.4%). 6 (40%) had normal CEA levels before and after surgery. Among these 6 cases, synchronous tumors were stratified Type I 1 (16.7%), Type II 0 (0%), Type III 4 (66.7%), Type IV 1 (16.7%).

**Table 5:** Relationship between CEA changes and clinicopathological features.

	Preoperative CEA elevated			Preoperative CEA normal		
	Postoperative cure	Postoperative stability	Postoperative recurrence	Postoperative cure	Postoperative stability	Postoperative recurrence
**Tumor diameter of MTC**	**6** (Patient n.6, 10, 13, 17, 26, 29)	**0**	**3** (Patient n.1, 25, 30)	**5** (Patient n.3, 14, 15, 20, 28)	**0**	**1** (Patient n.19)
≤1cm	**3** (Patient n.10, 13, 29)	**0**	**0**	**5** (Patient n.3, 14, 15, 20, 28)	**0**	**0**
1-2cm	**2** (Patient n.6, 17)	**0**	**1** (Patient n.1)	**0**	**0**	**0**
2-4cm	**1** (Patient n.26)	**0**	**1** (Patient n.30)	**0**	**0**	**1** (Patient n.19)
≥4cm	**0**	**0**	**1** (Patient n.25)	**0**	**0**	**0**
**Tumor invasion of MTC**	**0**	**0**	**2** (Patient n.1, 25)	**0**	**0**	**0**
**Lymph node metastasis of MTC**	**1** (Patient n.29)	**0**	**3** (Patient n.1, 25, 30)	**0**	**0**	**1** (Patient n.19)

Postoperative cure: Postoperative CEA was within the normal range, and there were no recurrent lesions;

Postoperative stability: Postoperative CEA was exceeded the normal range and remained at a high level, and there were no recurrent lesions;

Postoperative recurrence: Postoperative CEA was exceeded the normal range, and there were recurrent lesions.

### Follow-up and survival data

During the follow-up period, recurrence occurred in 4 patients (13.3%) after surgery (patient nos. 1, 19, 25, 30). Specifically, local recurrences (on the neck) occurred in 2 patients (patient no. 19 and no. 30) who required re-operation. Two patients (No. 1 and No. 25) died of distant metastasis (bone and liver). These last 2 patients were all male, BMI > 27kg/m^2^, Ctn and CEA were higher than normal, tumor diameter ≥2cm with N-metastases in central and lateral compartments. Patient no.1 had synchronous type I tumor and underwent total thyroidectomy + central N and bilateral N dissection. The proportion of N metastases was 67.1%. Patient No. 25 had a synchronous tumor type IV and underwent total thyroidectomy + central N and lateral N dissection. The N metastasis rate was 20%.

Two recurrent cases were all male, BMI in normal range. Ctn was higher than normal, CEA was slightly elevated in one case and within normal range in one case, tumor diameter was ≥2 cm. Patient no. 19 had a tumor type III and developed N-metastases in the central compartment. The proportion of N-metastases at the second surgery was 66.7%. Patient no. 30 had a IV type tumor and developed N-metastases in the central and lateral neck compartments. The N-metastasis rate at the first operation was 50%, at the second operation 26.7%.

## Discussion

### Pathogenesis

The pathogenesis of synchronous MTC/PTC is unclear. The collision theory states that the simultaneous occurrence of two tumors is merely coincidental and is based on the high incidence of PTC (11). The incidence of micro-PTC is high in the general population and about 80% of PTC with combined MTC are microscopic. Patients with MTC may incidentally develop a PTC at the same time (12). De facto, we report 26 (86.7%) synchronic micro-PTC/MTC. However, this hypothesis is difficult to accept in cases where the PTC was the dominant lesion. Further studies on the pathogenesis are needed.

### Clinical features and diagnosis

The age of patients ranged from 35 to 70 years, with a median of 55 years and a male-to-female ratio of 1.3:1. 90% of patients had no clinical symptoms. Similar to previous studies, it is difficult to make the diagnosis based on clinical presentation alone (13). Studies have shown that MTC in combination with PTC present predominantly hypoechoic or very hypoechoic nodules and microcalcifications on US, which is similar to the findings at our center (13). Patients mostly presented with hypoechoic nodules with poorly or well defined margins on US. Fine needle aspiration (FNA) usually diagnosed PTC or suspicious carcinoma. FNA cytology is thought to be of little diagnostic value in patients with MTC combined with PTC, as it is not easy to detect both pathological types of thyroid cancer (14–19). The results of FNA in our center were similar to previous studies. Ctn tests were performed in all patients and 70% had elevated values. fifteen patients were tested preoperatively for CEA, 60% had elevated levels. It has been suggested that abnormally elevated levels of calcitonin and CEA in laboratory tests are helpful in diagnosing MTC, but that it is difficult to diagnose MTC in combination with PTC.

Finally, sorting through the data, we found that MTC diameter basically did not change much from Type I to IV, but Ctn increased significantly.(**Figure 8**)

### Classification and pathology

We divided synchronous tumors into 4 types. The predominant subtype was type 3 (46.7%).

The majority of lesions were microscopic and most PTC were multifocal. Patients with a tumor diameter of 1-2 cm had higher than normal calcitonin and carcinoembryonic antigen levels, recurrence after surgery and a higher proportion of lymph node metastases. In patients with tumor diameters of 2 to 4 cm, calcitonin and carcinoembryonic antigen levels were mostly higher than normal, there were 2 cases of postoperative recurrence and a higher proportion of lymph node metastases. In patients with tumors > with a diameter of 4 cm, calcitonin and carcinoembryonic antigen levels may be higher than normal, with one case of postoperative recurrence and a higher proportion of lymph node metastases. According to our center’s data, patients with MTC combined with PTC who have a smaller tumor diameter, especially those with a diameter of < 1 cm, have lower calcitonin and carcinoembryonic antigen levels, a lower risk of postoperative recurrence, a lower proportion of lymph node metastases and a better prognosis. Immunohistochemical examination of MTC combined with PTC at our center showed a positive CT rate of 66.7%, a positive Syn rate of 80%, a positive CgA rate of 87.5%, a positive TTF-1 rate of 100% and a positive Congo red stain of 81.8%. Of note, the Tg positive rate was 44%, possibly related to the PTC being the dominant lesion and the MTC lesion < being 1 cm in size. Three patients were both CT and Tg positive. In patient No. 2, MTC and PTC were mixed lesions located on the left side with a diameter of 0.4 cm; in patient n.5, MTC and PTC were located on the left side with a diameter of 0.4 cm for the MTC and 0.1 cm for the PTC; and in patient No. 18, MTC and PTC were located on the left side with a diameter of 0.8 cm for the MTC and 0.2 cm for the PTC. The reason for the positive CT and Tg immunohistochemistry of the MTC lesions in these three patients could be due to the mixed lesions and the close proximity of the MTC and PTC lesions.

In our center, the rate of lymph node metastases was high in MTC combined with PTC, with patients with PTC as the dominant lesion more likely to have lymph node metastases, especially lymph node metastases in the central region, while patients with MTC as the dominant lesion had a larger maximum diameter of lymph node metastases and were more likely to have lymph node metastases in the lateral neck region. Some studies indicated a high rate of lymph node metastases in patients with MTC in combination with PTC, mostly involving MTC metastases, which differs from our center’s findings and needs further validation with big data. Some studies have pointed to the importance of genetic testing in the diagnosis of MTC and PTC. The occurrence of PTC is often caused by mutations in the BRAFV600E gene and the occurrence of MTC by mutations in the RET gene, and 20-40% of PTC also have abnormal expression of the RET gene. Although genetic testing has not been performed in our patients, it is equally important to confirm the importance of genetic testing in patients to ensure that they receive appropriate individualized diagnosis and treatment, their prognosis is improved and familial heritability is excluded.

### Follow-up

In patients with suspected MTC, a normal calcitonin level can be supplemented with a CEA test to improve the diagnosis. In the four patients with recurrence, the preoperative calcitonin level was above normal and the postoperative calcitonin decreased temporarily and increased again after some time. In three of the four patients with recurrence, the preoperative carcinoembryonic antigen levels were above normal and the postoperative carcinoembryonic antigen decreased, in one patient the carcinoembryonic antigen increased again after some time, and in one patient the carcinoembryonic antigen stabilized and increased slightly. In the other case, the carcinoembryonic antigen was at a normal level before, after and at the time of the second operation. Calcitonin and carcinoembryonic antigen levels affected the prognosis of patients with MTC combined with PTC to some extent, with calcitonin being more sensitive than carcinoembryonic antigen.

### Treatment and prognosis of patients with MTC in combination with PTC

Surgery is currently the most effective treatment for thyroid cancer, but the incidence of MTC in combination with PTC is low, and there is no consensus among national and international experts on the extent of surgery, nor are there recommendations in guidelines for the management of this disease. According to the American Thyroid Association guidelines, patients with MTC in combination with PTC should be operated on radically, as the aggressiveness of MTC is greater than that of PTC and the extent of surgery for MTC is greater than the extent of surgery for PTC. The basic extent of surgery is total thyroidectomy + bilateral lymph node dissection of the central group. The decision to perform lymph node dissection of the affected neck and bilateral cervical lymph nodes is based on the imaging suggestion and serum calcitonin level. At our center, 27 patients (90%) were operated on according to these guidelines, with one glandular lobe preserved in 3 patients (10%). According to the latest MTC guidelines, the extent of surgery was inadequate in these 3 patients, and recurrence occurred in one case, and the prognosis of the other 2 patients should be closely monitored.

The prognosis of the 30 patients with MTC combined with PTC in our center was good 4 patients suffered recurrence, 1 patient died of other diseases, and 25 patients survived to date, which is comparable to previous reports in the literature.

Since patients with MTC in combination with PTC have features of both tumors, they can be treated postoperatively with radioactive iodine therapy, endocrine therapy and targeted drug therapy. Some studies suggest that some patients with advanced disease can be treated with radiotherapy and chemotherapy to achieve local remission of the lesion, but big data are still needed to prove this. Patients’ prognosis is monitored by measurements of serum calcitonin, serum CEA, thyroglobulin and thyroid function, as well as ultrasound examinations of the neck and, if necessary, CT and PET-CT to determine whether patients have recurrent metastases.

### Limitations

Our study contains limitations inherent to a retrospective analysis. Further limitations may arise from unmeasured confounders and the inclusion of heterogeneous PTC sub-centers. In addition, US imaging, FNA, pathology, and lack of molecular data of the studied cohort have evolved and surgical techniques have changed.

## Conclusions

Synchronous PTC/MTC have been classified into 4 subtypes: (Type I) True mixed MTC/PTC, i.e. MTC and PTC are closely fused. (Type II) Collision MTC/PTC, i.e. tumors that meet at the same site, invade each other and appear as a single mass in the thyroid gland, i.e. MTC and PTC fuse. (Type III) Synchronous, anatomically separate tumors in the same thyroid lobe, i.e. separated by non-tumorous thyroid parenchyma. (Type IV) Synchronous tumors occurring in separate anatomical lobes or in the isthmus. This is probably the most numerous case series reported in the literature. Unique clinical and pathological aspects and outcomes are presented.

## Institutional review board statement

The study was conducted in accordance with the Declaration of Helsinki and approved by the Institutional Review Board (or Ethics Committee) of China-Japan Union Hospital Of Jilin University (protocol code:2022080401).

## Data availability statement

The original contributions presented in the study are included in the article/**Supplementary material**. Further inquiries can be directed to the corresponding author.

## Ethics statement

The studies involving human participants were reviewed and approved by China-Japan Union Hospital of Jilin University (protocol code: 2022080401). The patients/participants provided their written informed consent to participate in this study. Written informed consent was obtained from the individual(s) for the publication of any potentially identifiable images or data included in this article.

## Author contributions

All listed in the authorship of this paper meet all of the following conditions: (1) Authors make substantial contributions to conception and design, and/or acquisition of data, and/or analysis and interpretation of data; (2) Authors participate in drafting the article or revising it critically for important intellectual content; and (3) Authors give final approval of the version to be published. Conceptualization, GD, DZ, and HS; Formal analysis: DZ, HS, MY, XZ, GD, CW, KL, HW, HC, and CS; Data curation: DZ, HS, MY, XZ, GD, CW, KL, HW, HC, and CS; Methodology: GD, DZ, and HS; Writing original draft preparation, GD, DZ, and HS; Writing-review and editing, DZ, HS, MY, XZ, GD, CW, KL, HW, HC, and CS. All authors contributed to the article and approved the submitted version.

## Supplementary material

Supplementary Figure 1 Immunohistochemistry of patient n.6 that MTC and PTC were located closely. A:CgA(+); **(B)**CgA(+); **(C)** CT(+); **(D)** Syn(+);**(E)** Tg(-); **(F)** TTF-1(+).

Supplementary Figure 2 Immunohistochemistry of patient n.17 that MTC and PTC were located in the different tumor of ipsilateral lobe. A:Ck19(+); **(B)**CT(+); **(C)**TTF-1(+); **(D)** Ggh(+).

Supplementary Figure 3 Immunohistochemistry of patient n.26 that MTC and PTC were located in the different thyroid lobe respectively. **(A)** CD56(+); **(B)**CgA(+); **(C)** CT(+); **(D)** Galectin3(+);**(E)** Ggh(+);**(F)** Syn(+); **(G)** Tg(+); **(H)** TTF-1(+).

Supplementary Table 1 Lymph node metastasis of 15 patients.
