# Supplementary figures and images for: Thirty synchronous medullary and papillary thyroid carcinomas
Source: Front Endocrinol (Lausanne). 2023 Mar 31;14:1153248. doi: 10.3389/fendo.2023.1153248 (PMC10102529; doi:10.3389/fendo.2023.1153248)

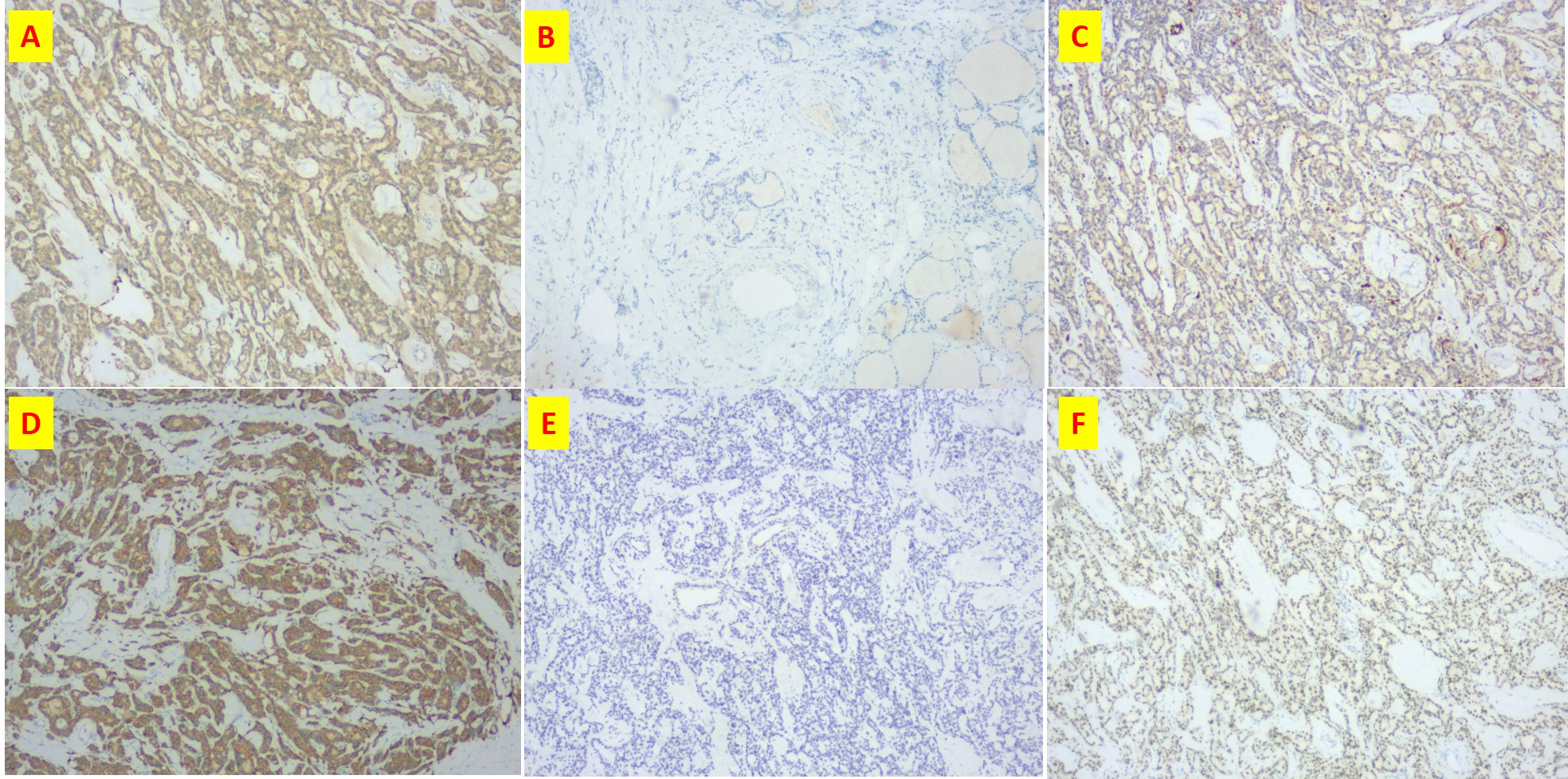

Supplement: Supplementary Figure 1 — Immunohistochemistry of patient n.6 that MTC and PTC were located closely. A:CgA(+); (B)CgA(+); (C) CT(+); (D) Syn(+);(E) Tg(-); (F) TTF-1(+). [file Image_1.jpeg]

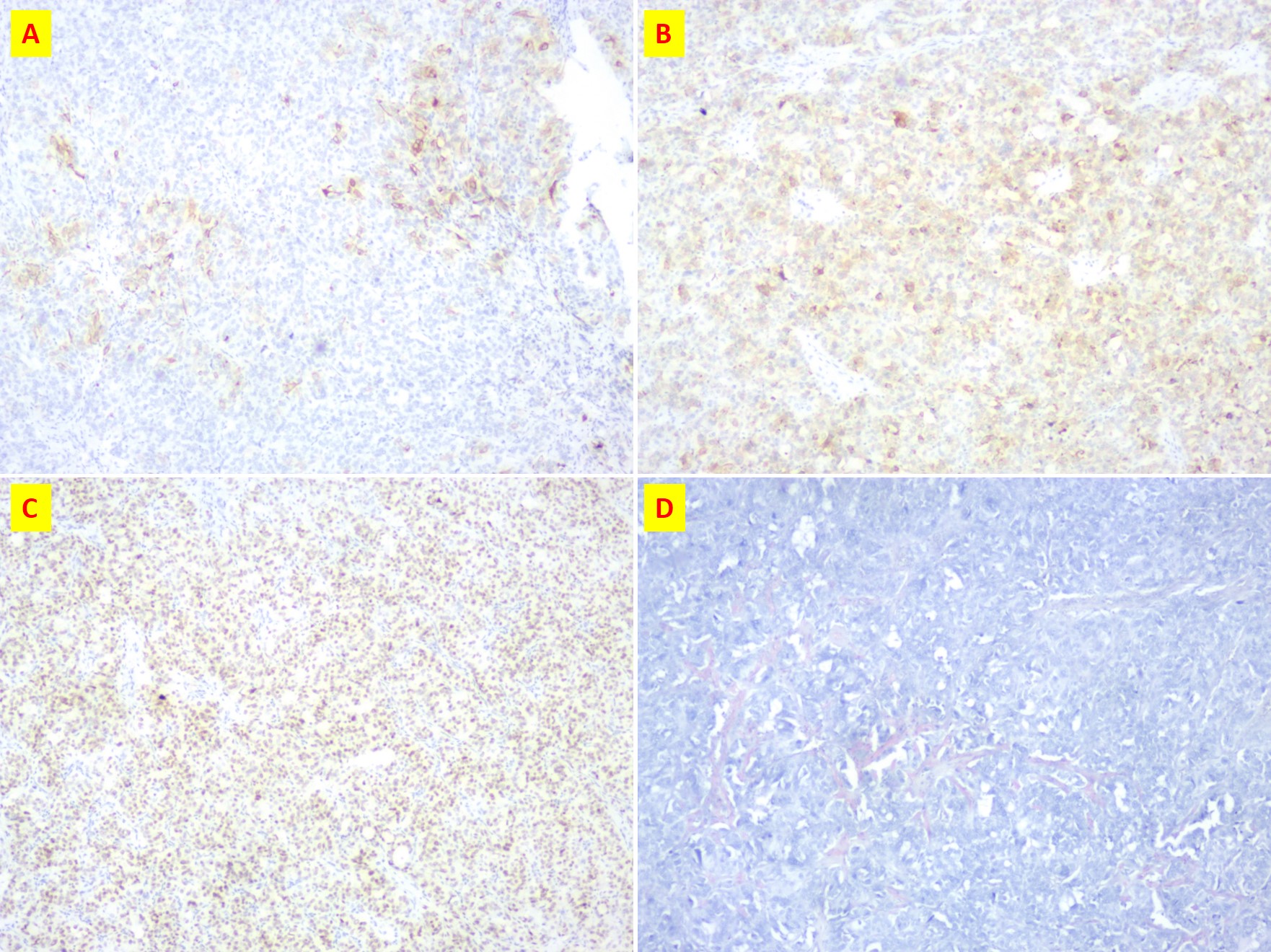

Supplement: Supplementary Figure 2 — Immunohistochemistry of patient n.17 that MTC and PTC were located in the different tumor of ipsilateral lobe. A:Ck19(+); (B)CT(+); (C)TTF-1(+); (D) Ggh(+). [file Image_2.jpeg]

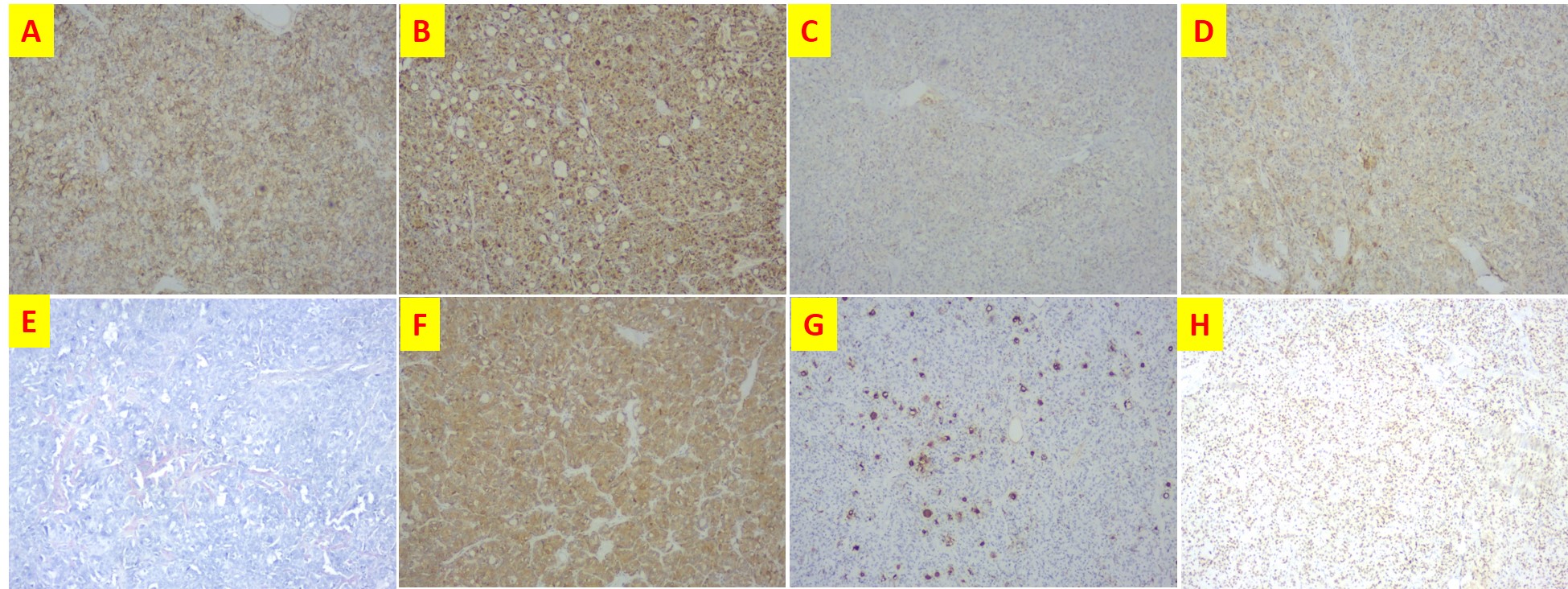

Supplement: Supplementary Figure 3 — Immunohistochemistry of patient n.26 that MTC and PTC were located in the different thyroid lobe respectively. (A) CD56(+); (B)CgA(+); (C) CT(+); (D) Galectin3(+);(E) Ggh(+);(F) Syn(+); (G) Tg(+); (H) TTF-1(+). [file Image_3.jpeg]
