# Supplementary material for: Thirty synchronous medullary and papillary thyroid carcinomas
Source: Front Endocrinol (Lausanne). 2023 Mar 31;14:1153248. doi: 10.3389/fendo.2023.1153248 (PMC10102529; doi:10.3389/fendo.2023.1153248)
Supplement: Supplementary Table 1 — Lymph node metastasis of 15 patients. [file Table_1.docx]

| Sup-Table 1 Lymph node metastasis of 15 patients | | | | | | | | | |
| --- | --- | --- | --- | --- | --- | --- | --- | --- | --- |
| Case | Age (years) | Gender | Tumor diameter (cm) | | Number of CLN metastases | | | Number of LLN metastases | |
|  |  |  | PTC | MTC | PTC | | MTC | PTC | MTC |
| **Type I** | | | | | | | | | |
| 1 | 51 | M | 2.0 | | 6 | 19 | | 9 | 21 |
| 2 | 30 | M | 0.4 | | 2 | NT | | NT | NT |
| 3 | 52 | F | 0.7 | | 1 | NT | | NT | NT |
| 4 | 76 | F | 11 | | 2 | NT | | NT | NT |
| **Type III** | | | | | | | | | |
| 8 | 44 | M | 0.6 | 2.5 | NT | | NT | 2 | NT |
| 11 | 49 | F | 0.7 | 0.4 | 2 | | NT | NT | NT |
| 15 | 41 | F | 0.6 | 0.6 | 1 | | NT | NT | NT |
| 19 | 49 | M | 0.5 | 3.5 | NT | | 2 | NT | NT |
| **Type IV** | | | | | | | | | |
| 22 | 57 | M | 0.15 | 2 | NT | | 2 | NT | 9 |
| 23 | 43 | M | 1.8 | 0.6 | NT | | NT | 2 | NT |
| 24 | 31 | F | 1.1 | 0.4 | 11 | | NT | 5 | NT |
| 25 | 68 | M | 0.2 | 4.5 | NT | | 1 | NT | 1 |
| 28 | 50 | F | 0.4 | 0.3 | 1 | | NT | NT | NT |
| 29 | 56 | M | 0.04 | 0.6 | NT | | 1 | NT | 2 |
| 30 | 22 | M | 0.1 | 2.5 | 2 | | 1 | NT | 2 |
| CLN: Central lymph node; LLN: Lateral lymph node; NT: No transfer; | | | | | | | | | |
